# Tartrate-resistant acid phosphatase 5b is a marker of osteoclast number and volume in RAW 264.7 cells treated with receptor-activated nuclear κB ligand

**DOI:** 10.3892/etm.2014.2071

**Published:** 2014-11-14

**Authors:** YUANDONG LV, GUIHUA WANG, WEIHONG XU, PINGHUA TAO, XIAOLING LV, YAZHEN WANG

**Affiliations:** Zhejiang Provincial Key Laboratory of Geriatrics, Zhejiang Hospital, Hangzhou, Zhejiang 310013, P.R. China

**Keywords:** RAW 264.7 cell, osteoclast, tartrate-resistant acid phosphatase 5b

## Abstract

Tartrate-resistant acid phosphatase 5b (TRACP 5b) has been used as a biomarker of bone resorption and cancer metastasis. TRACP 5b has also been suggested to be a reliable marker of osteoclast number. In this study, the correlation of TRACP 5b level and osteoclast-like cell number was investigated in RAW 264.7 cells treated with receptor-activated nuclear factor κB ligand (RANKL). RAW 264.7 cells were cultured with α-MEM containing RANKL (40 ng/ml) for 3, 5 and 7 days. Osteoclast formation and TRACP 5b levels were determined by TRACP staining, scanning electron microscopy and enzyme-linked immunosorbent assay. The RAW 264.7 cells that were not exposed to RANKL did not secrete TRACP 5b. RANKL induced the RAW 264.7 cells to differentiate into osteoclasts and to secrete TRACP 5b. The TRACP 5b level in the RAW 264.7 cells treated with RANKL was significantly correlated with the number and volume of osteoclasts (r=0.95 and r=0.92, respectively; P<0.0001). TRACP 5b is a good marker of RANKL-induced osteoclast formation in RAW 264.7 cells. TRACP 5b analysis may be used as an alternative to osteoclast counting *in vitro*.

## Introduction

Osteoclasts are important cells involved in bone resorption. They are multinucleated cells derived from hematopoietic cells (1–3). In the presence of receptor-activated nuclear factor κB ligand (RANKL) and macrophage colony-stimulating factor (M-CSF), hematopoietic precursor cells differentiate into mature osteoclasts, which are fused polykaryons arising from multiple monocytic cells (3,4).

Numerous *in vitro* models of osteoclast differentiation have been developed. It has been suggested that bone marrow cells (5), spleen cells (6) and blood monocytes (7) are able to differentiate into osteoclasts in the presence of certain specific factors. Tartrate-resistant acid phosphatase 5b (TRACP 5b) is one of several bone metabolic biomarkers that are specifically secreted by osteoclasts (8,9) and has been used as biomarker of bone resorption and cancer metastasis. Alatalo *et al* (10) and Rissanen *et al* (11) have shown that TRACP 5b is a good indicator of osteoclast number in mouse bone marrow-derived osteoclasts and human blood monocyte-derived osteoclasts.

The mouse macrophage cell line RAW 264.7 is a transfectable monocyte/macrophage cell line that retains the capacity to differentiate into osteoclast-like cells in the presence of RANKL (12,13). RAW 264.7 cells have been widely used and accepted as a cellar model of osteoclast formation and function in biology and pharmacology (14,15). However, to the best of our knowledge, no studies have investigated whether TRACP 5b can be used as an indicator of osteoclast number in RAW 264.7 cell-derived osteoclasts. In the present study, using the RAW 264.7 cell line, the association of TRACP 5b and the number of osteoclasts was investigated.

## Materials and methods

### RAW 264.7 cell culture

The RAW 264.7 mouse monocyte/macrophage cell line (American Type Culture Collection number, TIB-71) was obtained as a gift from Dr Hui Sheng (Department of Orthopedics and Traumatology, The Chinese University of Hong Kong, Hong Kong, China). The cells have the capacity to differentiate into osteoclast-like cells in the presence of RANKL. The cells were cultured in α-MEM containing 10% fetal bovine serum (FBS; Sigma-Aldrich, St. Louis, MO, USA), 100 U/ml penicillin and 100 μg/ml streptomycin at 37°C in a humidified atmosphere of 95% air and 5% CO_2_ with a change of medium every two days.

### TRACP staining

RAW 264.7 cells were cultured with α-MEM containing RANKL (40 ng/ml) for 3, 5 and 7 days with a change of medium every 2 days. The cells were fixed and stained for TRACP using an Acid Phosphatase, Leukocyte (TRAP) staining kit (catalog number, 387A; Sigma-Aldrich) according to the manufacturer’s instructions. Cells that were stained red were considered to be differentiated osteoclast-like cells and multinucleated cells were those comprising ≥3 nuclei. The number of TRACP^+^ cells was counted using Simple PCI imaging software (Compix Inc., Cranberry, PA, USA) by two individuals who were blinded to the previous treatment of the cells.

### Scanning electron microscope (SEM) examination

RAW 264.7 cells were cultured on glass slides with α-MEM containing RANKL (40 ng/ml) for 3, 5 and 7 days with a change of medium every two days. The RAW 264.7 cells were fixed using 4% paraformaldehyde and SEM analysis was conducted using a Hitachi S520 SEM (Hitachi Ltd., Tokyo, Japan). The slices were mounted on the SEM stub with carbon tape and carbon coated prior to analysis.

### TRACP 5b assay

RAW 264.7 cells were cultured with α-MEM containing RANKL (40 ng/ml) for 3, 5 and 7 days with a change of medium every two days and the supernatants on days 3, 5 and 7 were collected and stored at −80°C. A MouseTRAP™ enzyme immunoassay (EIA) kit (Immunodiagnostic Systems Ltd., Boldon, UK) was used in the determination of the level of TRACP 5b in the supernatants according to the instructions provided by the manufacturer. The intra-assay variation of the method for TRACP 5b was <6.5% and the inter-assay variation was <8%. The standard sample supplied by the kit was 2.0 (1.6–2.5) U/l, and the obtained result in our laboratory was 1.94 U/l.

### Statistical analysis

SPSS software, version 11.5 (SPSS Inc., Chicago, IL, USA) was used for statistical analysis. Data are represented as the mean ± standard deviation. The correlation of the TRACP 5b level and the number of osteoclasts was analyzed using Pearson correlation analysis. P<0.05 was considered to indicate a statistically significant difference.

## Results

### TRACP-positive cell formation

**Figure 1:**
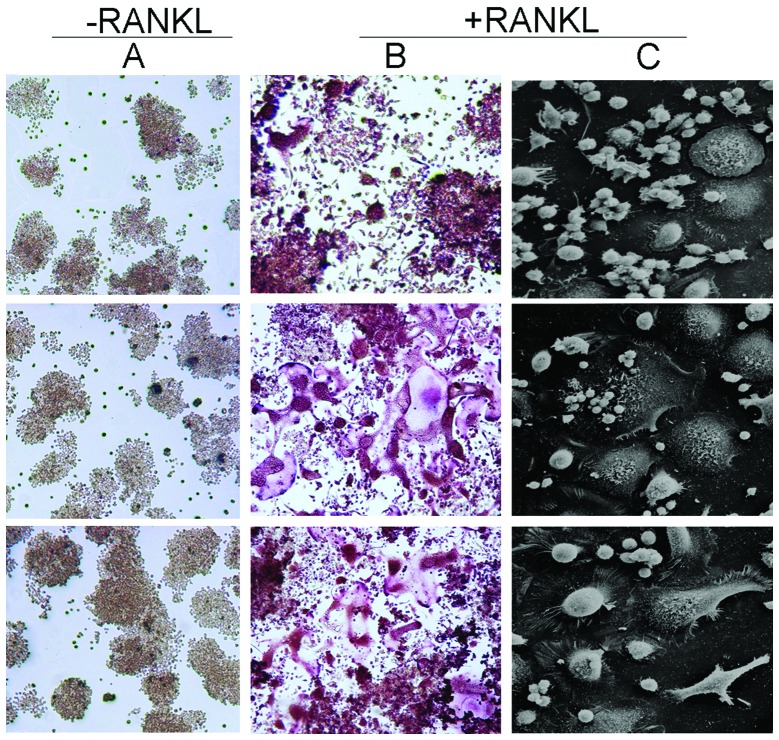
Tartrate-resistant acid phosphatase (TRACP) staining and scanning electron microscope (SEM) images of RAW 264.7 cells in the presence and absence of receptor-activated nuclear κB ligand (RANKL). TRACP staining of cells cultured for 3, 5 and 7 days (A) without and (B) with RANKL. (C) SEM imaging of cells cultured for 3, 5 and 7 days in the presence of RANKL. Top panels, 3 days; middle panels, 5 days; bottom panels, 7 days. For 1A and 1B, magnification, ×40. For 1C, magnification, ×400.

**Figure 2:**
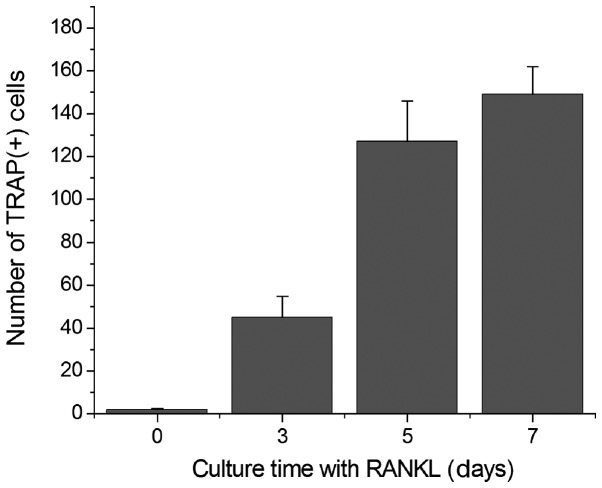
Number of tartrate-resistant acid phosphatase (TRACP)-positive cells among RAW 264.7 cells induced by receptor-activated nuclear κB ligand (RANKL). RAW 264.7 cells were cultured with α-MEM containing RANKL (40 ng/ml) for 3, 5 and 7 days. TRAP-positive cells were stained using a TRACP staining kit. TRACP-positive cells were counted using Simple PCI software.

The results of the TRACP staining assay indicated that RANKL induced the formation of osteoclasts from RAW 264.7 cells. No clearly TRAP^+^ cells were observed in the untreated RAW 264.7 cells (Fig. 1A). A number of multinucleated (≥3 nuclei) giant cells were formed in the RAW 264.7 cells that were treated with 40 ng/ml RANKL for 5 and 7 days (Fig. 1B). SEM showed the osteoclast-like cells were larger, darker, branched cells with a number of microvilli (Fig. 1C). Furthermore, the number of TRAP^+^ cells was determined. The results indicated that RANKL induced a marked increase in the number of TRAP^+^ cells (Fig. 2).

### TRACP 5b level

**Figure 3:**
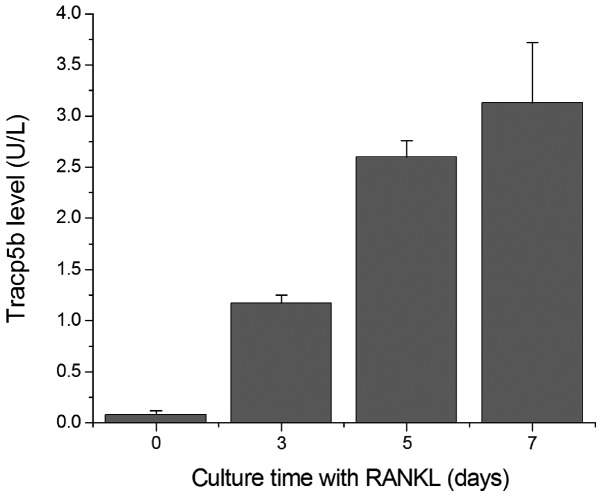
Tartrate-resistant acid phosphatase (TRACP) 5b levels in RAW 264.7 cells induced by receptor-activated nuclear κB ligand (RANKL). RAW264.7 cells were cultured with α-MEM containing RANKL (40 ng/ml) for 3, 5 and 7 days. The Tracp 5b levels in supernatants were determined using an enzyme immunoassay.

The TRACP 5b level in the RAW 264.7 cells was examined using an EIA kit. TRACP 5b was detected in the RAW 264.7 cells following treatment with RANKL for 3, 5 and 7 days, and the level of TRACP 5b increased as the culture time increased (Fig. 3).

### Correlation of TRACP 5b with osteoclast number and volume

**Figure 4:**
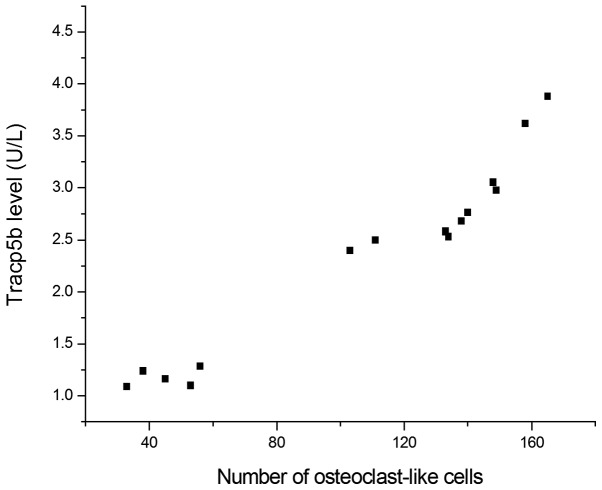
Correlation of tartrate-resistant acid phosphatase (TRACP) 5b level with the number of osteoclast-like cells.

**Figure 5:**
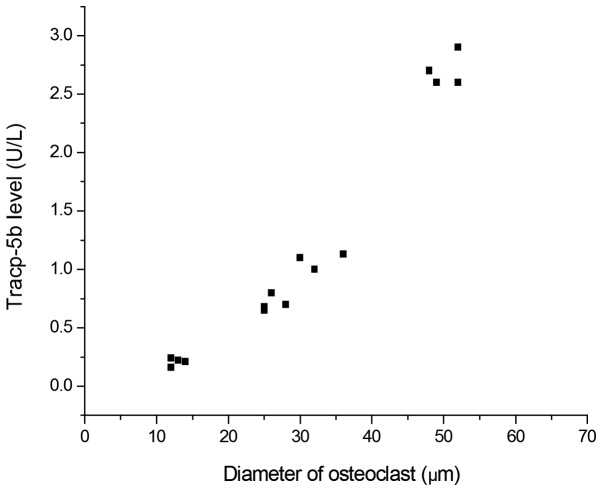
Correlation of tartrate-resistant acid phosphatase (TRACP) 5b level with the volume of osteoclasts formed.

The results of the correlation analysis, presented in Figs. 4 and 5, indicate that number and volume of osteoclast-like cells was significantly correlated with the level of TRACP 5b released into the medium (r=0.95 and 0.92, respectively; P<0.001).

## Discussion

In the present study, it was demonstrated that RANKL induced RAW 264.7 cell fusion to form mature osteoclasts. The level of TRACP 5b secreted into the culture medium by the RAW 264.7 cell-derived osteoclast-like cells was significantly correlated with the number of osteoclastic-like cells formed. These results suggest that TRACP 5b analysis may be used as an alternative to the microscopic counting of osteoclasts differentiated from RAW 264.7 cells with the presence of RANKL.

TRACP 5b has been used in the diagnosis of bone diseases (16,17), such as osteoporosis and bone metastasis, and for monitoring antiresorptive treatment (18). It has been suggested that TRACP 5b levels may reflect the status of bone resorption (9). TRACP 5b levels have been shown to correlate with the number of osteoclasts in patients with osteopetrosis (19) and renal bone disease (20). However, to the best of our knowledge, no studies have investigated the correlation of TRACP 5b levels with osteoclast number *in vitro*.

In the present study, it was observed that RAW 264.7 cells cultured with RANKL secreted TRACP 5b constitutively into the medium. The TRACP 5b level was correlated with the number of osteoclast-like cells. The data were consistent with the results observed by Alatalo *et al* (10). However, there was a certain difference in the findings. The plot of osteoclast number and TRACP 5b data in the present study formed a curve, not a straight line as was observed for the data provided by Alatalo *et al* (10) and Rassanen *et al* (11). The data appeared to fit a binomial curve. This may be due to the different cell type used in the present study. In addition, it is speculated that the TRACP 5b level may not be only associated with the osteoclast number, but also with osteoclast size. Big osteoclasts may secrete greater amounts of TRACP 5b into the culture medium compared with that secreted by smaller osteoclasts.

An increasing number of studies have adopted RAW 264.7 cell as a source of osteoclasts to study the effects of potential therapeutic agents on bone diseases (21,22) and to conduct osteoclast-related research (23–25). Thus, it would be of benefit to develop new methods for quantifying osteoclasts as an alternative to osteoclast counting using light microscopy. The data from the present study indicate that TRACP 5b immunoassay may be a fast and reliable method to replace time-consuming osteoclast counting.
